# Effect of Wendler glottoplasty on voice and quality of life of transgender women

**DOI:** 10.1016/j.bjorl.2021.06.010

**Published:** 2021-08-04

**Authors:** Mateus Morais Aires, Daniela de Vasconcelos, Jonia Alves Lucena, Adriana de Oliveira Camargo Gomes, Bruno Teixeira de Moraes

**Affiliations:** Universidade Federal de Pernambuco, Hospital das Clínicas, Departamento de Otorrinolaringologia–Cirurgia de Cabeça e Pescoço, Recife, PE, Brazil

**Keywords:** Transgender persons, Sex reassignment procedures, Surgery, Voice, Glottoplasty

## Abstract

**Objective:**

To investigate the effect of Wendler glottoplasty on voice feminization, voice quality and voice-related quality of life.

**Methods:**

Prospective interventional cohort of transgender women submitted to Wendler glottoplasty. Acoustic analysis of the voice included assessment of fundamental frequency, maximum phonation time formant frequencies (F1 and F2), frequency range, jitter and shimmer. Voice quality was blindly assessed through GRBAS scale. Voice-related quality of life was measured using the Trans Woman Voice Questionnaire and the self-perceived femininity of the voice.

**Results:**

A total of 7 patients were included. The mean age was 35.4 years, and the mean postoperative follow-up time was 13.7 months. There was a mean increase of 47.9 ± 46.6 Hz (*p* = 0.023) in sustained/e/F0 and a mean increase of 24.6 ± 27.5 Hz (*p* = 0.029) in speaking F0 after glottoplasty. There was no statistical significance in the pre- and postoperative comparison of maximum phonation time, formant frequencies, frequency range, jitter, shimmer, and grade, roughness, breathiness, asthenia, and strain scale. Trans Woman Voice Questionnaire decreased following surgery from 98.3 ± 9.2 to 54.1 ± 25.0 (*p* = 0.007) and mean self-perceived femininity of the voice increased from 2.8 ± 1.8 to 7.7 ± 2.4 (*p* = 0.008). One patient (14%) presented a postoperative granuloma and there was 1 (14%) premature suture dehiscence.

**Conclusion:**

Glottoplasty is safe and effective for feminizing the voice of transgender women. There was an increase in fundamental frequency, without aggravating other acoustic parameters or voice quality. Voice-related quality of life improved after surgery.

## Highlights

• There was an increase of 47 Hz in fundamental frequency after glottoplasty.
• There was no worsening in other acoustic measures and quality of voice.
• Voice-related quality of life improved after surgery.
• Glottoplasty was safe and effective for feminizing the voice of transgender women.

## Introduction

According to census data provided by the Instituto Brasileiro de Geografia e Estatística, the prevalence of transgender women is estimated to range from 1:35,000 to 1:50,000 in Brazil.^1^ The voice is an important marker of the transition process to the new gender identity of the transgender person. A voice that is incongruous with the female gender may be a limiting factor for fully exercising the social activities of a transgender woman.^2^ This could impact their emotional, social and vocational engagement, and might lead to avoidance behaviors and to a reduced quality-of-life.^3^ It has been demonstrated that modifying aspects of voice and communication related to gender reduces gender dysphoria and improves the mental health and quality of life of the transgender person.^4^

Transgender men generally achieve an acceptable male voice with androgenic hormone therapy.^5^ However, for transgender women, voice feminization is a great challenge, because hormonal treatment has no influence on the voice.^6^ In a survey with around 27,000 transgender women, 62% had either undergone or intended to undergo voice therapy (VT) or surgery to feminize their voice.^7^

The fundamental frequency (F0) is responsible for 50%–60% of vocal femininity.^8^ There seems to be a significant positive correlation between F0 and the perception of femininity, both by themselves and by listeners.^9^, ^10^ For cisgender people, the lower limit of F0 for a voice to be considered female is around 165 Hertz (Hz).^11^ The remaining femininity is determined by the formant frequencies, especially the first (F1) and second (F2), vocal quality and resonance. The female voice has higher formant frequencies, greater variability in the tonal curve, and is more breathy, soft, rhythmic and articulate.^12^

Voice therapy helps in acquiring female vocal behavior, modifying aspects of communication, such as intonation, sound quality, configuration and action of the laryngeal joints, and resonance of the vocal tract.^13^, ^14^ A meta-analysis has concluded that VT also has the potential of increasing F0, by about 31 Hz.^15^ Nonetheless, in uncontrolled situations, such as laughter, crying, sneezing and yawning, the male voice may appear. For 20% of patients seeking voice feminization, VT is not completely satisfactory, and surgery should be considered.^16^

The ideal surgery for vocal feminization should preserve the physiological phonatory function of the larynx, increasing the pitch without altering other acoustic parameters or vocal quality.^17^, ^18^ This has become a relatively recent challenge in phonosurgery. The surgical techniques described are based on three fundamentals: (1) increasing the tension of the vocal fold (VF), such as the cricothyroid approximation; (2) decreasing the VF mass, such as laser vaporization; and (3) decreasing the vibratory length of the VF, such as Wendler glottoplasty.^19^

Recent reviews have shown that the three strategies are effective in increasing the pitch, although more impressive results have been obtained through glottoplasty, considered to be the current practice.^15^ The endoscopic glottoplasty technique, described by Wendler^20^ in 1990, consists of de-epithelialization and suture of the anterior portion of the vocal folds, forming an anterior synechia, with the retropositioning of the anterior commissure and, thus, reduction of the vibrating length. The newly formed web does not interfere with breathing during rest or exercise, there is no need for an aesthetic scar and the increase in pitch seems to be long-lasting.^2^, ^21^, ^22^ The main disadvantage is that healing can be unpredictable, with the formation of granuloma or synechia longer or shorter than desired. In addition, due to the intentional formation of an area with fibrosis and scarring – known causes of dysphonia –, other acoustic parameters of the voice seem to worsen after glottoplasty. There may be a restriction of the maximum phonation time (MPT), frequency range and a worsening of voice quality.^23^

There is some hesitation in proceeding with phonosurgery after unsatisfactory improvement with VT due to the possibility of aggravating voice quality, a concern that appears in a document published by the World Professional Association of Transgender Health (WPATH).^4^ Therefore, the aim of this study is to describe the effect of glottoplasty in voice feminization, through measurement of F0, and in other acoustic parameters and quality of voice. Secondly, the objective is to quantify the impact of glottoplasty in voice-related quality of life, thereby enabling better assistance and information for transgender women who wish to feminize the voice.

## Materials and methods

### Ethical considerations

This study was conducted in accordance with the 1964 Helsinki declaration and was approved by the local ethics committee (number: 3,117,243). Informed consent was obtained from each patient.

### Study design and patients

This was a prospective interventional cohort, conducted at Hospital das Clínicas da Universidade Federal de Pernambuco, Recife, Brazil. The recruitment period was from March 2018 to October 2019. The population consisted of consecutive transgender women diagnosed with gender dysphoria, according to the criteria of the WPATH,^24^ monitored for at least 2 years by the hospital. This preoperative followup period in a multidisciplinary team is a prerequisite of the National Health System for gender-affirming surgery in public hospitals, in compliance with ordinance 2803/2013. Patients who wished to feminize their voice were submitted to weekly speech therapy for at least 3 months. In speech therapy, aspects were addressed related to vocal hygiene, breathing, resonance, vocal smoothing, speech rhythm, intonation, and fundamental frequency. After speech therapy, patients who maintained self-perception of male voice and low pitch according to the medical evaluation were eligible for the surgical procedure. The exclusion criteria established were low attendance to preoperative speech therapy (<50% of sessions), prior pitch-elevation surgery, postoperative follow-up period of less than 6 months or presenting with clinical or psychiatric comorbidity prohibiting surgical treatment.

### Pre- and postoperative assessment

In the immediate preoperative period, all patients provided demographic data, answered the questionnaires Trans Woman Voice Questionnaire (TWVQ) and Self-perceived Femininity of the Voice (SpFv), as described below, and underwent laryngostroboscopy and voice assessment using acoustic analysis, auditory-perceptual assessment. The postoperative followup protocol consisted of periodic consultations (1 week; 1, 3 and 6 months) for anamnesis and laryngostroboscopy exam in order to assess the anatomical and functional status of the larynx and to identify possible complications. For the purpose of comparative analysis, after 6 months of followup, all patients were submitted to the same voice assessment performed preoperatively. For acoustic analysis of the voice, samples were recorded digitally in a quiet environment using a directional microphone (Auricular Karsect HT-2) and filtering and noise reduction equipment (Andrea PureAudio™ USB-AS). The microphone was placed 4.0 cm from the mouth at an angle of approximately 45°, with the participant in a sitting position at a 90° angle, and hands resting on the legs. Voice recordings were stored for later acoustic analysis independently using Voxmetria® and Fonoview® (CTS Play). The voice of each patient was recorded in an individual sound file and anonymously labeled. These samples were randomized for subsequent auditory-perceptual assessment of voices.

We obtained the MPT for the vowel/a/after taking a deep breath, at a comfortable volume and pitch (three attempts, the longest phonation time was included in the study). Fundamental frequency of sustained/e/(F0/e/), formant frequencies (F1 and F2), jitter and shimmer were obtained through emission of the sustained vowel/e/. Frequency range and Speaking Fundamental frequency (SF0) were computed through the analysis of CAPE-V protocol phrases.^25^ The auditory-perceptual assessment of the voice through Hirano grade, roughness, breathiness, asthenia, and strain (GRBAS) scale^26^ was conducted blindly and independently by three speech-language therapists specialized in voice, who were non-participants in the research. Fleiss’ Kappa reliability test was applied. The most common assessment for each parameter (at least 2 of the 3 speech-language therapists) was that included in the study. This scale has five different determined parameters: G (Grade), R (Roughness), B (Breathiness), A (Asthenia) and S (Strain). A four-point rating scale (0 = normal, 1 = slight, 2 = moderate, 3 = severe) was used for each parameter.

Voice-related quality of life was measured using the TWVQ, translated into 13 languages and validated for Brazilian Portuguese.^1^, ^27^ It is a Patient-Reported Outcome Measure (PROM) which was developed with the aim of providing a reliable measurement of self-reported vocal function and influence of the voice on transgender women individuals’ daily lives.^27^ Higher self-ratings of voice femininity are associated with lower TWVQ scores and lower voice-related impact to quality of life.^28^ It contains 30 questions (scores from 1 to 4 each, with a total score of 30–120 points) that can be organized into three factors (anxiety and avoidance, vocal identity, and vocal function), and may be used as a pre- and post-treatment measure by comparing total scores.^29^, ^30^

To quantify the perception of the voice gender by the patient herself, the SpFv was used. The SpFv was measured with a visual analogue scale ranging from 0 (the most male voice the transgender could imagine) to 10 (the most female voice the transgender could imagine).^31^

### Surgical technique

**Figure 1:**
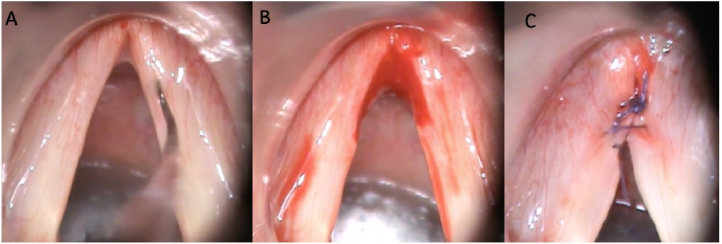
Surgical technique. (A) Initial aspect. (B) De-epithelization of the anterior third of the membranous vocal folds. (C) Final aspect, with two Vicryl 4.0 sutures.

All patients were underwent glottoplasty under general anesthesia with orotracheal intubation by the same team of otolaryngologists (the authors MMA and BTM), using the same surgical technique as described by Wendler^20^ (Fig. 1). We exposed the larynx through a rigid suspension laryngoscope and de-epithelialized, using cold curved microscissors, the anterior third of the membranous portion of the vocal folds, including free edge and superior and inferior sides. We preserved the vocal ligament in all cases. The de-epithelialized portion was sutured together with 2 or 3 sutures, using 4.0 Vicryl with a 15 mm gauge needle. The desired synechia size was 33% of the anteroposterior length of the membranous portion of the VF.

During the postoperative period, patients were recommended to remain in absolute vocal rest for 14 days, and relative vocal rest for additional 14 days. Patients were prescribed with proton pump inhibitor (omeprazole 80 mg/day, for 30 days), antibiotic (amoxicillin–clavulanate 1750 mg/day, for 7 days) and antitussive if necessary (codeine syrup). As all patients had undergone speech therapy protocol during the preoperative period, postoperative speech therapy was not performed until the last measurement of acoustic parameters described in this research.

### Statistical analysis

Statistical analysis was performed using SPSS 23.0 (IBM, Armonk, NY). Descriptive statistics were presented as mean ± Standard Deviation (SD) with a 95% Confidence Interval (CI) for continuous variables, and as frequencies (%) for categorical ones. Statistical significance was compared using a Mann–Whitney Signed Rank test, because of the small number of patients and the non-normal distribution of the variables. Results were considered statistically significant when the p-value was less than 0.05.

## Results

**Figure 2:**
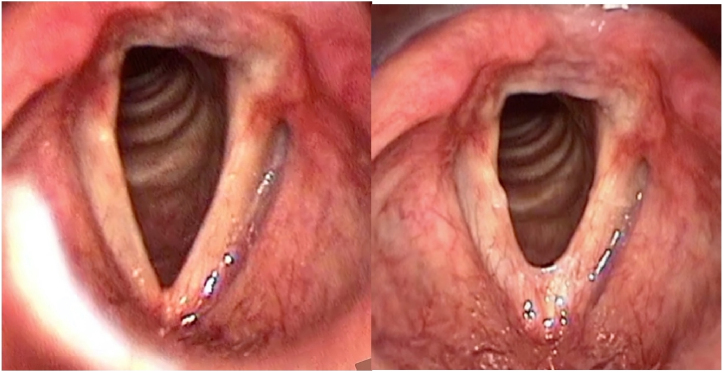
Laryngeal image before (left) and 3 months after glottoplasty (right) in inspiration, patient #5.

A total of 7 patients were included. The mean age was 35.4 years (range 27–45 years). The mean time of multidisciplinary monitoring at the service before undergoing glottoplasty was 4.1 years (range 2–7 years). The mean number of weekly sessions of preoperative speech therapy was 24.6 (range 13–54 sessions), with a mean period of 5.7 months. Three (43%) patients underwent glottoplasty in isolation, while 4 (57%) underwent glottoplasty with chondrolaryngoplasty to reduce laryngeal prominence, as reported in a previous case series.^32^ The mean duration of the surgery was 1:10 h. Fig. 2 presents pre- and postoperative laryngoscopy for patient #5.

### Acoustic analysis of voice

The fundamental frequency data are described in Table 1. In relation to F0/e/, there was a mean increase of 47.9 ± 46.6 Hz (*p* = 0.023) after glottoplasty. In the individual analysis, 6 (86%) patients presented a considerable increase in F0/e/. In the SF0 assessment, there was a mean increase of 24.6 ± 27.5 Hz (*p* = 0.029) after glottoplasty. In the individual analysis, 5 (71%) patients presented a considerable increase in SF0.

**Table 1:** Pre- and postoperative fundamental frequency.

Subject	F0/e/			SF0		
	Pre-op	Post-op	Variation	Pre-op	Post-op	Variation
1	184	214	+30	176	197	+21
2	111	243	+132	131	210	+79
3	153	190	+37	137	155	+18
4	133	130	−3	138	133	−5
5	128	158	+30	153	179	+26
6	134	224	+90	146	178	+32
7	121	140	+19	134	135	+1
**Mean (SD)**	**137.7 (24.1)**	**185.6 (43.8)**	+47.9 (46.6)*	**145.0 (15.5)**	**169.6 (29.9)**	+24.6 (27.5)*

F0/e/, fundamental frequency of sustained/e/; SF0, speaking fundamental frequency; Pre-op, preoperative; Post-op, postoperative; SD, standard deviation.

* Statistically significant at *p* < 0.05.

With regard to MPT, jitter, shimmer, frequency range, F1 and F2, there was no statistical significance in the pre- and postoperative comparison (n = 7) (Table 2).

**Table 2:** Acoustic and aerodynamic assessments.

	Pre-op, mean (SD)	Post-op, mean (SD)	*p*-Value
MPT, seconds	12.5 (2.1)	18.9 (14.1)	0.794
Jitter, %	1.32 (2.4)	0.96 (1.3)	0.397
Shimmer, %	15.34 (8.4)	7.65 (3.3)	0.073
Frequency range, ST	8 (2.7)	8 (3.4)	0.425
Frequency range, Hz	75.0 (33.6)	82.0 (40.6)	0.352
F1, Hz	560 (122)	532 (184)	0.849
F2, Hz	1907 (163)	1711 (467)	0.441

Pre-op, preoperative; Post-op, postoperative; SD, standard deviation; MPT, maximum phonation time; ST, semitones; Hz, Hertz; F1, first formant; F2, second formant.

### Perceptual analysis of voice quality

The mean score of each component of the GRBAS scale, blindly assessed by three speech therapists (*κ* = 0.61, substantial inter-rater reliability), did not change in the 6 months postoperative evaluation (n = 7) (Table 3). Individually, 4 (57%) patients presented no difference and 3 (43%) demonstrated an improvement in the GRBAS scale score after glottoplasty. All patients had some degree of roughness in the first 3 postoperative months.

**Table 3:** Perceptual analysis of voice (GRBAS scale).

	Pre-op, mean (SD)	Post-op, mean (SD)	*p*-Value
Grade	0.71 (0.75)	0.29 (0.75)	0.275
Roughness	0.57 (0.53)	0.14 (0.38)	0.200
Breathiness	0.29 (0.75)	0.29 (0.75)	0.952
Asthenia	0	0	0.952
Strain	0	0	0.952

Pre-op, preoperative; Post-op, postoperative; SD, standard deviation.

### Voice-related quality of life

TWVQ decreased following surgery from 98.3 ± 9.2 (range 80–107) to 54.1 ± 25.0 (range 32–80) (*p* = 0.007).

### Self-perceived femininity of voice

The mean SpFv score increased from 2.8 ± 1.8 (range 1–5) to 7.7 ± 2.4 (range 3–10) after glottoplasty (*p* = 0.008).

### Complications

**Figure 3:**
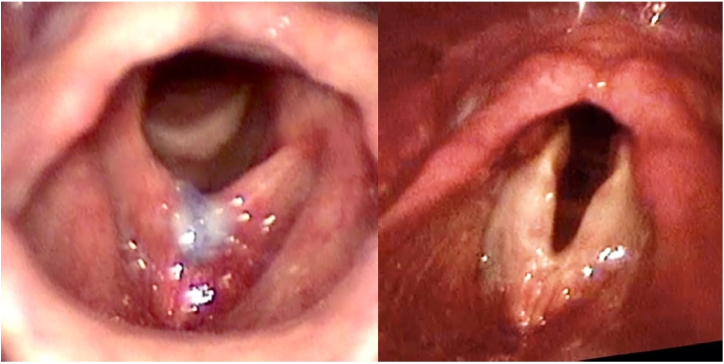
Laryngeal image of granuloma pre- (left) and post-treatment (right), patient #7.

Patient #4 developed premature suture dehiscence with no anterior web formation (n = 1; 14%) and failed to raise the pitch of her voice (a drop of 3 Hz in F0), which required a revision surgery. Patient #7 developed a postoperative granuloma (n = 1; 14%), treated with inhaled corticosteroid (Fig. 3). There was no major complication such as dyspnea due to hypertrophic synechia.

## Discussion

The aim of this study was to analyze the acoustic and voice quality changes in transgender women undergoing Wendler glottoplasty.

Fundamental frequency is the most important parameter in gender perception of a voice. There is a linear correlation between the vocal pitch and gender perception of voice.^9^, ^33^ We observed a significant increase in the fundamental frequency, both in the sustained vowel (47.9 Hz) and during speech (24.6 Hz). Recent meta-analysis has defined a mean increase in F0 after glottoplasty of 78 Hz, a higher value than in our study.^23^ We believe that these different findings are due to the fact that most of the authors included in the meta-analysis performed varying degrees of postoperative speech therapy before the acoustic analysis of the voice.^18^, ^22^, ^34^, ^35^ In isolation, speech therapy has the potential of increasing 31 Hz in F0,^15^ which may have overestimated the specific power of glottoplasty to raise the pitch in these studies. As all patients in our study had already undergone a long preoperative speech therapy protocol before surgery, they did not undergo speech therapy in the postoperative period. We believe, therefore, that the increase in F0 observed in our study is exclusively due to glottoplasty, thereby making them reliable. A similar situation occurred with Chang et al.,^36^ who only performed 1 or 2 speech therapy sessions during the postoperative period and reported a mean increase in SF0 by 20 Hz. Individually, glottoplasty was successful in satisfactorily raising the SF0 in 5/7 patients (71%) in our sample, a result identical to that of Chang et al.^36^

In patients with borderline F0 (145–165 Hz), formant frequencies, related to the vocal timbre and determined by the size and shape of the vocal tract, are used to define the gender of the voice. F1 is inversely correlated with the tongue height and F2 changes by the advancement of the tongue in the horizontal plane.^37^ In the female voice, the values of the formant frequencies are higher, especially F1.^11^, ^16^, ^38^ In our sample – since glottoplasty does not alter the anatomy of the vocal tract – the values of F1 and F2 did not change with surgery, remaining in the range attributable to the male gender.^39^, ^40^ Thus far, only speech therapy is able to modify the resonance of the vocal tract and increase the formant frequencies.^41^ It is postulated, therefore, that, due to the male phenotype of the vocal tract (and male F1, F2 and resonance), the lower limit of the F0 for the voice of transgender women being recognized as female is around 180 Hz.^42^, ^43^ Taking into account the need for a higher F0 for adequate feminization of the voice, some authors believe that shortening the VF by 33%, as originally described by Wendler, may be insufficient for transgender women to achieve a female voice and have chosen to increase the proportion of the synechia. Lately, the proportion of up to 50% of the length of the membranous portion of the VF has been used.^17^, ^44^, ^45^

With the formation of the anterior synechia and reduction of the vibratory area of VF by glottoplasty, voice production could be less effective, resulting in a lower MPT and a lower frequency range of the voice, i.e., less possibility of pitch variation, thereby resulting in a voice considered less natural.^44^ However, other than F0, no other acoustic parameter had a statistically significant change after surgery in this study. This suggests that Wendler glottoplasty does not result in objective worsening of the voice as a general rule, although the number of patients in this study is limited. It is possible that larger studies with greater numbers of patients may find different results. A meta-analysis compiling five studies showed that glottoplasty might slightly decrease MPT and frequency range.^23^

There is little data that correlates other acoustic parameters and gender attribution to the voice. Shimmer has already been moderately negatively correlated with listener femininity ratings.^46^ We were unable to achieve this correlation in this study due to the limited number of patients. Additional research is needed to clearly describe the relationship between other acoustic parameters and vocal femininity, defining the measurements that should be assessed in the context of glottoplasty.

Voice quality is adequately assessed in a simple manner using the classical GRBAS scale of Hirano. While perceptual measures are inherently subjective, they may detect changes in the voice that are not fully recognized in objective acoustic measurement. With specific reference to glottoplasty, the perception of roughness or breathiness after surgery has been described, although the results are controversial.^22^, ^23^, ^31^, ^34^ Although we found variable degree of roughness in the voice of all patients in the first 3 months after the procedure, this dysphonia was transient since there was no worsening on the GRBAS score after 6 months of surgery. This suggests that Wendler glottoplasty does not necessarily worsen voice quality, with the exception of the initial healing period.

In addition to measuring acoustic parameters as indicators of surgical success, it is reasonable to quantify it through PROMs. These are more individualized and comprehensive than acoustic measurements or surgical satisfaction rates. Ultimately, PROMs translate the individual’s perception of their voice and communication, and their improvement is therefore the ultimate goal of the surgery. Although the minimal clinically important difference for TWVQ has not been previously determined for transgender patients undergoing pitch elevation surgery, higher self-ratings of voice femininity are associated with lower TWVQ scores and a lower voice-related impact to quality of life and may be used as a pre- and post-treatment measure by comparing total scores.^29^ This has only been the second study to use TWVQ (or TVQ^MtF^) as a PROM after glottoplasty. As in the study by Chang et al., in our sample the TWVQ decreased significantly following surgery from 98 to 54 points, which suggests an improvement in the voice-related quality of life after glottoplasty.^36^ Furthermore, in our sample, the mean SpFv score increased significantly from 2.8 to 7.7 after glottoplasty, demonstrating the perception of greater vocal femininity by the patient herself.

In relation to complications, we observed 1 granuloma (14%), which was clinically resolved with inhaled corticosteroid, and 1 suture dehiscence (14%). Similar rates were obtained in previous cohorts, which related suture dehiscence to non-compliance with vocal rest or smoking.^8^, ^17^, ^21^, ^31^^,^^45^ Although we recommended vocal rest for all operated patients, we did not assess whether it had been adhered to, so we are unable to make this association. Other complications mentioned by other authors, such as anterior glottic gap, were not observed in our sample. There was also no major complication, such as dyspnea due to hypertrophic synechia.^31^

This study has some limitations and weaknesses. The main limitation is the small sample. Multivariate regression could not reasonably be performed in this study due to the sample size. On the other hand, the result of an increase in F0 with statistical significance – the main outcome evaluated in the study – associated with the improvement of subjective and objective variables (auditory-perceptual evaluation, acoustic parameters, voice-related quality of life and self-perception of voice) after glottoplasty, corroborate the thesis that these results are not the consequence of sampling error. In addition, a longer followup period would determine if the pitch is sustained in the long term, a possible advantage of glottoplasty over other surgical techniques, evidence that cannot be ratified with a followup of just 6 months.

We believe that the most important point of this work is the prospective analysis of F0 and voice quality without postoperative speech therapy, making it possible to attribute the results exclusively to glottoplasty in a more reliable manner. In addition, we used a specific and validated PROM for the studied population to quantify the impact of surgery on voice-related quality of life.

## Conclusion

Glottoplasty was a safe and effective procedure for feminizing the voice of transgender women. There was an increase in fundamental frequency, without aggravating other acoustic parameters or voice quality. The voice-related quality of life, measured by TWVQ, improved after surgery.

## Conflicts of interest

The authors declare no conflicts of interest.
